# Heterogeneous associations of health expenditure, environmental pollution, and economic growth on life expectancy in BRICS economies

**DOI:** 10.3389/fpubh.2026.1767163

**Published:** 2026-02-24

**Authors:** Mohammad Ridwan, Zulfiquar Ali Antor, Afsana Akther, Jeremy Ko, Hossein Ali Fakher, Chun Kai Leung, Wai-Kit Ming

**Affiliations:** 1 Department of Economics, Noakhali Science and Technology University, Noakhali, Bangladesh; 2 Center for Comparative and International Studies, ETH Zurich, Zurich, Switzerland; 3 Department of Accounting, Ayandegan University, Tonekabon, Iran; 4 Global Society and Sustainability Lab, The University of Hong Kong, Hong Kong, Hong Kong SAR, China; 5 Department of Infectious Diseases and Public Health, City University of Hong Kong, Hong Kong, Hong Kong SAR, China

**Keywords:** BRICS economies, economic growth, healthcare expenditure, life expectancy, sustainability

## Abstract

This study investigates the associations between economic growth, healthcare expenditure, environmental pollution, urbanization, trade openness, and life expectancy in BRICS economies from 2000 to 2024 using a distribution-sensitive panel framework. Quantile regression is applied to capture heterogeneity across different levels of life expectancy, supported by robustness checks using PCSE, DKSE, and F-GLS estimators, and validated through panel cointegration and dependence tests. The results show that healthcare expenditure is positively associated with life expectancy, while the relationships for economic growth and pollution are mixed and vary with development stage. Urbanization exhibits both supportive and adverse associations depending on infrastructure capacity and environmental pressure, and trade openness generally relates to lower life expectancy. These findings suggest that policy responses should be tailored rather than uniform across countries. Strengthening the efficiency of healthcare systems, coordinating industrial growth with pollution management, and adopting environmental and health safeguards in trade and urban policy can help BRICS governments align economic expansion with sustainable improvements in population well-being.

## 1 Introduction

The BRICS countries—Brazil, Russia, India, China, and South Africa—represent some of the world’s most dynamic emerging economies, yet they exhibit substantial disparities in life expectancy even amid global improvements in mortality coverage (1). This persistent heterogeneity raises critical questions about how economic growth, healthcare investment, and environmental pressures interact to shape human longevity. Globally, life expectancy at birth has risen from 66 years in 1990 to 73 years today (2, 3), but among the BRICS, differences remain stark: while the average life expectancy in China exceeds 77 years, South Africa’s is approximately 64 years (4, 5). These contrasts mirror pronounced disparities in prosperity—India’s GDP per capita remains below USD 2,500, whereas Russia’s surpasses USD 12,000 (6, 7). Collectively, the BRICS account for roughly 40% of global CO₂ emissions (8) and spend between 3 and 9% of GDP on health (9, 10). These variations make BRICS an especially compelling empirical context for investigating how prosperity, health systems, urban dynamics, and environmental burdens jointly influence lifespan outcomes. Economic prosperity remains a key determinant of longevity. Numerous studies confirm that GDP per capita, healthcare expenditure, and social spending positively affect life expectancy (2, 7, 11). Demographic variables—fertility, education, and human development—are also tightly associated with income and survival outcomes (6, 12). Health spending is particularly decisive: cross-country evidence shows that higher investment in healthcare correlates with longer lifespans (13). According to WHO, W (14), between 1995 and 2013, Brazil, China, South Africa, and Russia increased their health expenditures as shares of GDP by 3.0, 2.0, 1.5, and 1.2%, respectively, while India’s share slightly declined. Yet, the effectiveness of these resources depends not only on spending volume but also on governance quality and health system efficiency.

Environmental conditions—especially carbon emissions and air pollutants—are another major determinant of longevity. A growing body of research demonstrates that sustained exposure to CO₂ and PM 2.5 significantly reduces life expectancy (15, 16). However, such adverse effects are mediated by socio-economic and institutional factors: countries with stronger economies and higher health investments may partially offset environmental degradation through adaptive infrastructure and medical resilience (17). For instance, in China, a 1% annual rise in PM 2.5 exposure has been associated with a 2.94% increase in household healthcare costs (18). Similarly, urbanization exerts mixed effects—moderate urbanization improves access to services and infrastructure, enhancing life expectancy, whereas unmanaged urban growth amplifies pollution and health risks (19–21). Trade openness further adds complexity: while global market integration can boost income and fund social services, it can also accelerate industrialization and pollution, particularly in developing economies (22–25). Together, these interconnections highlight the necessity of an integrated framework linking economic, environmental, and social determinants of health. Despite extensive global research, the BRICS context remains undertheorized and empirically underexplored. These economies are uniquely positioned at the crossroads of rapid growth, demographic transformation, and environmental stress, yet systematic investigations into how these forces jointly determine longevity are scarce. Existing studies often rely on global or continental averages, masking intra-group heterogeneity. Furthermore, conventional mean-based econometric methods inadequately capture how determinants of life expectancy vary across different points in the distribution—understanding which factors matter most in lower- or higher-life-expectancy contexts remains an open question.

This study is designed to address these gaps by (i) evaluating how economic growth, healthcare expenditure, environmental quality, urbanization, and trade openness are associated with life expectancy in BRICS economies, both individually and jointly; (ii) investigating whether these relationships vary across different points of the life-expectancy distribution; and (iii) applying a quantile regression framework to capture potential heterogeneity in these associations. In doing so, the study seeks to strengthen empirical understanding of the development–health–environment nexus in major emerging economies and to support more evidence-informed policy discussion on balancing growth, sustainability, and population well-being. To operationalize these objectives, the analysis follows a structured empirical strategy. Stationarity is examined using panel unit-root tests, including Levin–Lin–Chu (LLC), Cross-Sectionally Augmented IPS (CIPS), and Augmented Dickey–Fuller (ADF). Long-run relationships among the variables are evaluated using the Pedroni panel cointegration test. The core estimation approach employs quantile regression to analyze distributional differences, complemented by robustness estimators such as Panel-Corrected Standard Errors (PCSEs), Driscoll–Kraay Standard Errors (DKSEs), and Feasible Generalized Least Squares (F-GLS), along with Dumitrescu–Hurlin panel causality testing to explore directional linkages.

This study aims to make two main contributions. First, it positions the BRICS economies within the broader global discussion on health, environment, and development by examining life expectancy through a multi determinant framework. Second, it applies an analytically richer empirical approach that is designed to capture nonuniform effects of economic, environmental, and social factors across different points of the life expectancy distribution, patterns that are often not visible in traditional mean based analyses. The remainder of the paper is organized as follows: Section 2 reviews the theoretical and empirical literature on life expectancy determinants; Section 3 presents the dataset, variables, and methodology; Section 4 reports and discusses the empirical results; Section 5 presents policy implications and conclusions; and Section 6 outlines limitations and directions for future research.

## 2 Literature review

The literature reviewed in this study was selected through a structured thematic approach focused on empirical and theoretical contributions related to life expectancy determinants. Priority was given to peer reviewed journal articles that examine the roles of economic growth, health expenditure, environmental quality, urbanization, and trade openness using panel or time series methods. The review combines recent empirical evidence with earlier foundational studies to ensure both topical relevance and conceptual continuity. Studies were included based on methodological rigor, variable comparability, and policy relevance to multi country or emerging economy contexts, particularly those comparable to BRICS economies.

### 2.1 GDP and life expectancy

The relationship between economic growth and life expectancy has been widely examined in development and health economics, with early cross country research documenting a strong and persistent income–health gradient (26–28). These foundational studies show that higher income levels are generally associated with longer lives, although the strength of this link varies across stages of development. More recent work applies nonlinear and panel methods to capture heterogeneity and structural differences across countries. Evidence from Vietnam indicates that education and healthcare reforms play a central role in longevity gains, with gender gaps shifting over development phases (29). Using broader welfare indicators, Barbier and Mensah (30) find that while environmental health improvements matter, GDP per capita still delivers larger average returns for life expectancy, especially in low and middle income economies. Yeboah et al. (1) show that growth supports longevity but may simultaneously worsen ecological conditions, with delayed benefits from renewable energy. Sector focused results also support a positive income effect, as Akter et al. (31) report that income, aquaculture, and food production raise longevity in exporting nations. Macroeconomic instability works in the opposite direction: Hataminia and Mohammadzadeh Asl (32) link inflation and unemployment to lower life expectancy. Large panel evidence confirms that GDP per capita improves longevity while pollution reduces it, and that health spending effects vary by income group (33). Crisis based analysis further shows that recessions increase mortality and inequality (34). Overall, growth supports life expectancy, but distributional, environmental, and macroeconomic risks shape outcomes.

*H*1: Economic growth increases life expectancy in the BRICS countries.

### 2.2 Health expenditure and life expectancy

Health spending is well-known as a major factor in determining life expectancy, although the extent of influence differs across nations and situations. Foundational research in health economics established that health investment functions as a form of human capital that influences longevity outcomes (35, 36). According to Singh et al. (37), Central Europe and the Baltic states report that health expenditures are generally converged with life expectancy; however, there is still a difference in government versus non-government expenditure. Adebayo et al. (38) employ quantile-on-quantile regression on the United States and find that urbanization and CO₂ emissions have negative influences on life expectancy, whereas income and health expenditure have positive effects. In BRICS economies (2000–2019), Kaur et al. (39) determine that government spending on health leads to increases in GDP and GNI per capita, but the effect on life expectancy is not that significant. Moreover, Khan et al. (40) demonstrate in Pakistan (2000–2020) that the health expenditure and sustainable development goals are integrated with life expectancy and there is a short-run causal relationship. The study by Bétila (41), which uses the System-GMM in 48 African countries (2000–2018), illustrates that ICT use improves the effectiveness of health expenditure that leads to the increase of life expectancy and the decrease in mortality. Lastly, Boundioa and Thiombiano (42) apply the threshold analysis (1996–2018) to show that the quality of governance must have critical levels of improvement in life expectancy by spending on public health. Overall, the literature provides consistent evidence that higher health expenditure contributes to improvements in life expectancy, reflecting the critical role of healthcare investment in enhancing population well-being.

*H*2: Health expenditure increases life expectancy in the BRICS countries.

### 2.3 Air pollution and life expectancy

A large body of environmental health research has established that exposure to air pollution is closely linked to higher mortality and reduced longevity across countries and regions (43, 44). These early studies laid the foundation for later cross country analyses showing that pollution related health risks remain significant even after accounting for income and demographic factors. More recent work confirms that air pollution interacts with economic and development conditions in shaping life expectancy outcomes. Evidence from Saudi Arabia shows that CO₂ emissions and PM2.5 concentrations are associated with declines in both life expectancy and healthy life expectancy, although policy reforms under Vision 2030 have partly moderated these effects (15). Broad panel results for developing economies also report a statistically significant negative effect of CO₂ emissions on longevity (45). Country studies offer mixed short and long run patterns. In Malaysia, carbon emissions reduce life expectancy in the short run, while health expenditure improves it (46). In contrast, results for Sub Saharan Africa suggest that GDP growth, industrial activity, and emissions can move positively with life expectancy, challenging the Environmental Kuznets Curve hypothesis (47). Distribution sensitive estimates for GCC countries show that emission effects remain negative across quantiles (48). Mediation evidence from Indonesia links emissions to lower human development outcomes despite gains in income and schooling (49). Additional African and SAARC panel studies confirm that emissions generally reduce life expectancy, whereas renewable energy and urban development can offset part of the damage (50, 51).

*H*3: Air pollution decreases life expectancy in the BRICS countries.

### 2.4 Urbanization and life expectancy

The relationship between urbanization and health has long been discussed in demographic and development research, with early studies noting that urban growth can generate both infrastructure advantages and environmental or social risks (52). Urbanization influences life expectancy through multiple channels, including access to healthcare, education, sanitation, and employment, while also increasing exposure to congestion and pollution. Recent empirical work highlights these mixed effects. Using Chinese data from 1990 to 2022, Zhang et al. (53) show that education, urbanization, and green growth improve life expectancy when environmental pressures are controlled, whereas CO₂ emissions reduce longevity. Cross country African evidence based on multilevel models indicates that unemployment and HIV prevalence widen life expectancy gaps, but urbanization helps moderate these disparities by improving service access (54). Micro level evidence also suggests that the health effects of aging depend partly on urban context, income, and lifestyle conditions, confirming a mediating role for urban environments (55). However, results are not uniformly positive. Quantile based analysis in highly polluted countries finds that poor air quality and unmanaged urban expansion are associated with lower life expectancy, while governance quality and inclusive development improve outcomes (56). Additional distribution sensitive evidence from Ghana shows that the effects of urbanization vary across health and income levels, producing unequal longevity outcomes (57). Taken together, the literature indicates that urbanization affects life expectancy in uneven ways, depending on environmental management and institutional quality.

*H*4: Urbanization decreases life expectancy in the BRICS countries.

### 2.5 Trade openness and life expectancy

Earlier globalization and health research indicates that trade integration can influence population health through several channels, including income growth, technology transfer, and environmental exposure (58). Empirical evidence from different regions generally finds a positive long run association between trade openness and life expectancy, although short run and indirect effects often vary. For Pakistan, cointegration and causality results show that trade openness and foreign direct investment support longer life expectancy over time (59). Similar findings appear in Nigeria, where trade credit linked to export and import activity contributes to longevity gains, highlighting the role of trade financing (60). Panel causality evidence from Latin America also suggests that trade openness significantly improves life expectancy, especially in less advanced economies with higher tax burdens (61). Country studies provide more nuanced results. For China, regime switching models indicate a dual effect in which trade raises income and health outcomes but may also increase emissions that offset part of the benefit (23). Malaysian evidence likewise shows that exports and imports are positively associated with lifespan through their connection with economic growth (62). Broader African panel estimates using System GMM link welfare and longevity improvements with stronger trade and port performance (63). Additional ARDL results for China confirm that trade openness, public spending, and human capital support life expectancy in the long run, with weaker short run effects (64). Overall, most studies report a positive but context dependent relationship between trade openness and longevity.

*H*5: Trade openness increases life expectancy in the BRICS countries.

### 2.6 Conceptual framework synthesis

**Figure 1:**
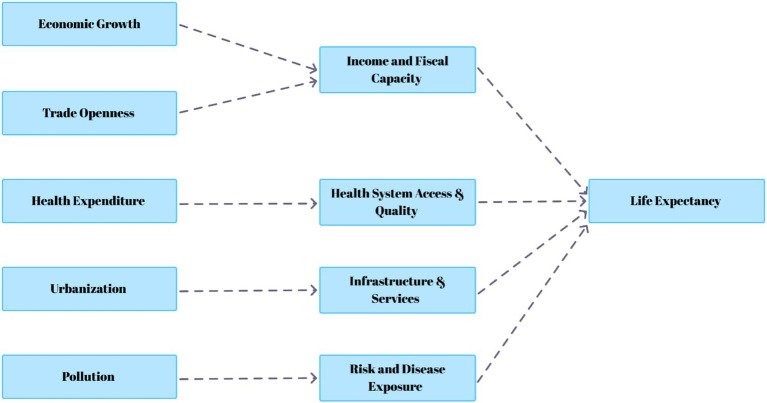
Conceptual framework of the study.

The accumulated evidence indicates that the existing literature suggests that life expectancy is shaped by a network of interdependent economic, social, environmental, and structural determinants rather than by any single factor in isolation. Economic growth and trade openness affect longevity primarily through their influence on income levels, structural transformation, and fiscal capacity to finance public health systems. Health expenditure operates more directly by improving access to medical services, preventive care, and treatment effectiveness. Environmental pollution contributes to higher disease burden and mortality risk through sustained exposure effects, while urbanization alters health outcomes by reshaping infrastructure availability, service delivery, and living conditions, with results varying by planning quality and institutional effectiveness. These channels interact and often reinforce one another through both direct and indirect transmission mechanisms. Viewed jointly, this evidence aligns with an extended health production function framework in which longevity outcomes depend on multiple coordinated inputs. This integrated perspective motivates the multivariable empirical specification adopted in the following section. Figure 1 presents the conceptual framework of the study.

### 2.7 Literature gap

Although previous studies present useful information about what determines life expectancy, such as economic growth, health spending, environmental pollution, urbanization, and openness to trade (15, 23, 29, 59), there are still several significant gaps. The majority of research is focused on individual nations or states, especially in the Western world, Africa, or Asia, and not on the BRICS economies, which have peculiarities in the form of high rates of economic growth, serious environmental issues, and heterogeneous demographics. Also, current research is mainly based on specific mean econometric models, which cannot quantify different heterogeneous effects of such determinants on various population groups or life expectancy lines. Economic, health, environmental, urban, and trade interactions are usually considered individually, overlooking the complex interactions between them that determine the endpoint of longevity. Conditional distributions of life expectancy are also seldom analyzed but are especially important in the emerging economies, where income inequality, uneven urbanization, and environmental strains are widespread across space and social ranks. Lastly, although a part of the studies examines causality of variables, there are few studies that use powerful panel models that consider cross-sectional dependence, heterogeneity, and dynamic interactions all in the case of BRICS countries. As a result, a methodological study that combines various determinants and takes distributional heterogeneity into account is highly lacking, which creates a significant gap of knowledge about subtle factors in life expectancy in these rapidly changing economies.

## 3 Data and methodology

### 3.1 Data description

Table 1 presents the variables used to examine the health–economy–environment nexus in the BRICS economies. The selection of indicators was guided by the principles of data completeness, comparability, and reliability across all five countries over 2000–2024, ensuring both cross-sectional coherence and temporal consistency within a balanced panel framework. Life expectancy at birth (LLE) serves as the dependent variable and is drawn from the World Bank’s World Development Indicators, which provide a standardized and internationally comparable measure. LLE is commonly used as an overall indicator of population health because it reflects general mortality conditions and summarizes the average number of years a newborn is expected to live, given current age-specific death rates. Although it does not capture every dimension of health status, it offers a broad and consistent benchmark that incorporates the combined influence of factors such as basic living conditions, healthcare access, and environmental exposure. Its definitional stability and long-term availability also make it well suited for comparative and trend analysis across emerging and developing economies (65, 66).

**Table 1:** Data description.

Variable	Description	Unit of measure	Source
LLE	Life expectancy at birth refers to the average number of years a newborn is expected to live, assuming that the current mortality rates at the time of birth remain constant throughout the individual’s lifetime.	Total (years)	World Bank (92)
LGDP	Gross domestic product per capita represents as a proxy of level of economic development is the average economic output or income per person, calculated by dividing the total value of goods and services produced within an economy over a specific period by the total population of that territory.	(constant 2015 US$)	World Bank (92)
LHE	Government health expenditure per capita, measured in current U. S. dollars, represents the government’s annual spending on healthcare goods and services consumed during the year, excluding capital expenditures such as buildings, equipment, information technology, and vaccine stockpiles for emergencies or outbreaks.	per capita (current US$)	World Bank (92)
LCO2	Carbon dioxide (CO₂) emissions per country-year represent the total amount of CO₂ released into the atmosphere from the burning of fossil fuels, industrial processes, and other human activities within a country during a specific year.	Total (Mt CO2e)	World Bank (92)
LUP	Urban population refers to the number of people residing in areas classified as urban by national statistical authorities. The data are compiled and adjusted by the United Nations Population Division.	(% of total population)	World Bank (92)
LTOP	Trade openness is measured as the sum of a country’s exports and imports expressed as a percentage of its gross domestic product (GDP), indicating the degree of integration with the global economy.	(% of GDP)	World Bank (92)

Among the explanatory variables, GDP per capita (LGDP)—measured in constant 2015 U. S. dollars—was operationalized as a standardized indicator of economic performance and average living standards. Its use allows meaningful comparison across countries and over time by expressing real output per person in inflation-adjusted terms, thereby minimizing price and currency distortions. Government health expenditure per capita (LHE)—reported in current U. S. dollars—captures annual public spending on healthcare goods and services actually consumed within the year, excluding capital investments such as buildings, equipment, or information technology. This operationalization isolates recurrent expenditure that directly reflects the fiscal capacity of governments to sustain health service delivery and coverage (67). Carbon dioxide emissions (LCO₂)—expressed in million metric tons—were selected as the sole environmental indicator because CO₂ data provide complete temporal and cross-country coverage within the World Development Indicators and ensure consistent measurement across all BRICS members. This selection enables comparable long-series estimation of environmental pressure associated with industrial activity and energy consumption, while alternative pollution indicators, such as PM₂.₅ concentrations or water contamination, lack continuity and definitional uniformity for the full study period (68).

Urban population (LUP)—the percentage of people living in areas classified as urban by national statistical authorities—was used to capture the demographic and structural transformation associated with industrialization and expansion of services. This indicator was chosen because it is harmonized by the United Nations Population Division within the World Development Indicators, ensuring consistent definitions of “urban” across countries and through time. Such harmonization minimizes potential measurement bias arising from national classification differences and enhances the comparability of results within a multi-country framework. Trade openness (LTOP)—defined as the sum of exports and imports expressed as a percentage of gross domestic product—was operationalized as a standard ratio widely used in cross-country economic research (69). This specification scales trade volume relative to economic size, allowing meaningful comparison among economies with different output levels and structural compositions. It also provides a transparent and scale-adjusted indicator of external economic integration that aligns with the study’s macro-panel design. Together, these operational definitions and transformations underpin a consistent and balanced dataset, allowing for elasticity-based interpretation within the logarithmic specification and making it possible to examine how economic development, fiscal capacity, urbanization, environmental pressure, and trade integration jointly influence life expectancy across the BRICS economies.

All series are transformed into natural logarithmic form to reduce heteroskedasticity and allow elasticity based interpretation of coefficients. The data are obtained from the World Development Indicators database, which provides internationally comparable cross country statistics on health and development indicators. The final dataset forms a balanced panel covering five BRICS countries over the period 2000 to 2024, with 25 annual observations per country and 125 total observations. Variable definitions were checked for consistency across countries and years, and after verification no missing observations remained, so no interpolation or imputation procedures were required. This balanced structure supports stable multi model and distribution based estimation.

Table 2 indicates definite differences in the mean values and dispersion of the indicators. The mean of life expectancy is high (4.236), and the standard deviation is low (0.092), with the result meaning that there are similar health outcomes between BRICS. Both carbon emissions and GDP per capita indicate high standard deviation (1.144) and mean (7.241), respectively, as well as economic and environmental disparities. The mean (5.577) of health expenditure is large with a broad range of the government expenditures. Conversely, the means of urbanization and openness to trade are moderate (4.054 and 3.751) with low standard deviations (0.359 and 0.288) and have more stable trends. Such differences explain why quantile regression is more suitable because average-based procedures would fail to capture such variations.

**Table 2:** Descriptive statistics.

Variable	Obs	Mean	Std. dev.	Min	Max
LLE	125	4.236	0.092	3.987	4.359
LGDP	125	8.527	0.754	6.629	9.482
LHE	125	5.577	1.165	2.917	7.294
LCO2	125	7.241	1.144	5.848	9.546
LUP	125	4.054	0.359	3.32	4.478
LTOP	125	3.751	0.288	3.096	4.221

Source: Authors’ calculation.

Table 3 presents the connection of life expectancy and all the independent variables. The lifespan is positively correlated with GDP (0.304), health expenditure (0.189), carbon emissions (0.538), and negatively correlated with urbanization (−0.163). The data demonstrate that economic expansion, enhanced healthcare finance, and urbanization positively influence longevity, whereas trade exposure may introduce adverse health or environmental consequences. The positive association between life expectancy and emissions reflects the outcomes of the initial stages of industrialization in emerging economies.

**Table 3:** Pairwise correlation.

Variables	(1)	(2)	(3)	(4)	(5)	(6)
(1) LLE	1.000					
(2) LGDP	0.304	1.000				
(3) LHE	0.189	0.935	1.000			
(4) LCO2	0.538	−0.053	−0.260	1.000		
(5) LUP	0.163	0.924	0.910	−0.383	1.000	
(6) LTOP	−0.412	−0.111	−0.157	0.203	−0.212	1.000

The relatively high correlations among GDP, health expenditure, and urbanization indicate some degree of interdependence among these variables and raise the possibility of multicollinearity affecting coefficient estimates. We accounted for this issue by applying robust estimation methods—specifically, Panel-Corrected Standard Errors (PCSE), Driscoll–Kraay Standard Errors (DKSE), and Feasible Generalized Least Squares (F-GLS)—in order to produce consistent standard errors and reliable inference in the presence of correlated regressors, heteroskedasticity, and cross-sectional dependence. Furthermore, the quantile-regression approach focuses on conditional distributions of life expectancy rather than mean effects, which helps lessen the sensitivity of estimates to potential multicollinearity among strongly related predictors. These combined steps help ensure that the relationships identified are stable and reflective of underlying structural patterns rather than distortions from correlated explanatory variables.

### 3.2 Theoretical framework

A significant economic model in the field of health economics is the Grossman (35) Model, which explains the interaction between health and economic behavior. It considers health as stock capital, which individuals invest to improve well-being and productivity. Although health is a natural process that worsens with age, it can be retained or enhanced by using investments like medical care, healthy lifestyles, and education, which are explained by the model. Building on this view, Smith and Dunt (70) proposed the health production function that incorporates both the health expenditures and non-health inputs as determinants of health outcomes. In the recent past, Shaari et al. (71) added health investments to their model, emphasizing that they enhance life expectancy. Equation (1) reflects the Grossman health function.

Health output=f(health inputs) (1)

Extending the model to include economic, environmental, and social variables, the health output (life expectancy at birth) can be modeled using government spending on health and government variables:

$$LE_{\mathrm{it}}=f\;(\mathrm{GDP},\mathrm{HE},\mathrm{CO}2,\mathrm{UP},\mathrm{TOP})$$ (2)

In Equation (2) LE = life expectancy which is a dependent variable and GDP = gross domestic product, CO2 = carbon dioxide emission, UP = urbanization and TOP = trade openness are the independent variables.

Equation (3) represents the logarithmic format.

$$\begin{array}{l} \mathrm{LL}E_{\mathrm{it}}=\beta_{0}+\beta_{1}\mathrm{LGD}P_{\mathrm{it}}+\beta_{2}\mathrm{LH}E_{\mathrm{it}}+\beta_{3}\mathrm{LC}O_{2\mathrm{it}}+\\ \beta_{4}\mathrm{LU}P_{\mathrm{it}}+\beta_{5}\mathrm{LTO}P_{\mathrm{it}}+\varepsilon_{\mathrm{it}}\;\;\;\; \end{array}$$ (3)

We use the logarithmic form of representation in this work to stabilize the variation of data and linearize nonlinear relationships. In addition, it enables us to examine coefficients as elasticity effects or percentages.

### 3.3 Econometric approach

The study resorts to a rigorous panel econometric methodology to examine the various impacts of economic growth, healthcare spending, air pollution, urbanization, and open trade on life expectancy in BRICS countries during 2000–2024. It commences by analyzing the cross-sectional dependence (CSD) and homogeneity of slope to consider interdependencies and structural differences across the BRICS countries. The CSD test as suggested by Pesaran (72) is a test that is used to determine whether the shocks in one country have an influence on the other in the panel, and this is mostly applicable to highly intertwined economies. The slope homogeneity test (73) is used in testing the homogeneity of the slope coefficients across nations to assist in deciding whether heterogeneous estimators are appropriate. Having confirmed the cross-sectional correlations and heterogeneity, panel unit root tests are then used to test the stationarity of the variables. Three tests are used in complement: Levin, Lin, and Chu (LLC) (74), Cross-sectionally Augmented IPS (CIPS) (75), and Augmented Dickey-Fuller (ADF), which together confirm that all variables are integrated of order one, I(1). Subsequently, the panel cointegration test by Pedroni is performed in order to reveal the long-term equilibrium relationship between life expectancy, GDP, health expenditure, emissions, urbanization, and trade openness (76, 77). The findings affirm that these variables are co-moved over the long run among BRICS countries.

The study uses quantile regression to investigate asymmetric and heterogeneous effects of economic, health, and environmental factors on life expectancy (78). In contrast to traditional mean-based estimators, quantile regression permits the study of the way the impacts of explanatory factors differ across the conditional distribution of life expectancy. The method is especially applicable to the BRICS situation, where nations are at varying levels of growth and where they have significant differences in terms of income, urbanization, and health infrastructure. The approach identifies the subtle effects of GDP, healthcare spending, emissions, urbanization, and trade openness on nations with low, medium, and high rates of life expectancy by examining the effects at various quantiles (e.g., lower, median, and upper tails).

Mathematically, the conditional quantile functionality is written as for Equation 4:

$$Q_{{\mathrm{LLE}}_{\mathrm{it}}}(\tau \;|X_{\mathrm{it}})=\alpha_{i(\tau )}+X_{\mathrm{it}}^{\prime }\;\beta (\tau ),0<\tau <1$$ (4)

where $\;Q_{2{\mathrm{LLE}}_{\mathrm{it}}}(\tau \;|X_{\mathrm{it}})$ denotes the τ-th conditional quantile of life Expectancy, $\alpha_{i(\tau )}$ captures country fixed effects, and β(τ) represents the vector of slope parameters that vary across quantiles.

The estimation is obtained by solving the minimization problem as shown in Equation 5:

$$\hat{\beta }(\tau )=\arg\;\min_{\beta }\sum_{i=1}^{N}\;\sum_{t=1}^{T}\;\rho_{\tau }({\mathrm{LLE}}_{\mathrm{it}}-\alpha_{i(\tau )}-X_{\mathrm{it}}^{\prime }\beta (\tau ))$$ (5)

Where $\rho_{\tau }(\mu )=\mu (\tau -I\{\mu <0\})$ is the quantile loss function.

Lastly, models are robust by use of various estimation methods. The possible heteroskedasticity, autocorrelation, and cross-sectional correlation problems are addressed by Panel-Corrected Standard Errors (PCSEs) (79), Driscoll-Kraay Standard Errors (DKSEs) (80), and Feasible Generalized Least Squares (F-GLS) (81), which prove the results to be stable. In an effort to investigate the causation direction further, the Dumitrescu-Hurlin (D-H) panel causality test is used (82). This approach determines both unidirectional and bidirectional causal relationships between variables and offers insights on the dynamic relationships between life expectancy, economic growth, health expenditure, urbanization, emissions, and trade openness in BRICS countries. This intensive panel econometric system as a whole makes the results robust and policy-relevant.

Several specification considerations were taken into account when designing the empirical strategy. First, potential endogeneity cannot be fully ruled out, as reverse relationships may exist between health outcomes and macroeconomic variables. Due to data and instrument constraints in a multi country macro panel setting, the estimates are interpreted as conditional associations rather than strict causal effects. Second, correlation diagnostics indicate moderate to high associations among some regressors, particularly GDP, health expenditure, and urbanization. To reduce inference bias from cross sectional dependence, heteroskedasticity, and residual correlation, multiple robust estimators including PCSE, DKSE, and F-GLS are applied. Third, CO₂ emissions may partly capture industrialization and development intensity rather than pure environmental damage, and coefficient interpretation is therefore made with this structural channel in mind. Quantile regression is selected because the primary objective is to examine distributional heterogeneity rather than average effects.

It should be noted that the quantile regression framework is designed to identify heterogeneous conditional relationships across the distribution of life expectancy and is not, by itself, a causal identification strategy. While robustness estimators and panel causality tests are employed to examine directional patterns, the reported coefficients should be interpreted as distribution specific associations. Accordingly, policy implications are drawn with appropriate caution and focus on structural relationships rather than definitive causal magnitudes.

## 4 Results and discussions

Table 4 examines the presence of cross-sectional dependence among variables in the BRICS panel. The significantly elevated CD-statistics (*p* < 0.01) seen across all variables, with the exception of trade openness (LTOP), confirm the transmission of shocks between countries, reflecting the economic and environmental interconnections within BRICS. This dependency elucidates the applicability of second-generation approaches, such as CIPS and quantile regression, which address cross-sectional correlation in this context. The independence of LTOP suggests that trade policies can be relatively distinctive to individual nations, even amidst global integration.

**Table 4:** CSD test.

Variable	CD-stat	*p*-value
LLE	14.682	0.000***
LGDP	13.759	0.000***
LHE	14.116	0.000***
LCO2	9.8	0.000***
LUP	15.342	0.000***
LTOP	−1.07	0.285

*p < 0.1, **p < 0.05, ***p < 0.01. Source: Authors’ calculation.

The slope homogeneity results in Table 5 indicate that the D and modified D statistics (*p* < 0.01) are significant, signifying that the relationships among variables are heterogeneous. It suggests that economic growth, healthcare expenditure, and environmental degradation significantly affect life expectancy among BRICS nations. This form of heterogeneity warrants the application of quantile regression, as the assumption of a uniform slope in traditional models would obscure country-specific variations and yield imprecise average results.

**Table 5:** Slope homogeneity test.

Test statistic	Delta	*p*-value
	6.991***	0.000
Adj.	8.239***	0.000

*p < 0.1, **p < 0.05, ***p < 0.01. Source: Authors’ calculation.

The outcomes of stationarity are presented in Table 6, determined by the LLC, CIPS, and ADF tests. These variables are all integrated of order one, I(1), indicating that their first differences are stationary. This indicates that the data series exhibit stochastic tendencies and possess long-run equilibrium relationships. The evidence of order I(1) among variables contradicts the results of panel cointegration tests. The findings guarantee that the regression results will not be misleading and that long-term causal processes can be properly established.

**Table 6:** Stationary test (LLC, CIPS, ADF).

Variable	LLC		CIPS		ADF		
	I(0)	I(1)	I(0)	I(1)	I(0)	I(1)	Order
LLE	−3.3138***	−6.7939***	−3.475***	−3.212***	6.8869	49.0291***	I(1)
LGDP	−4.3360***	−4.9760**	−1.841	−2.470**	21.9832***	23.1678***	I(1)
LHE	−3.9412***	−6.4810***	−2.306*	−3.857***	15.6982	32.7162***	I(1)
LCO2	−3.8231***	−5.8328***	−0.480	−2.994***	13.4379	32.9788***	I(1)
LUP	−30.4828***	−0.7881	1.155	−2.726***	143.7904***	1.7484	I(1)

*p < 0.1, **p < 0.05, ***p < 0.01. Standard errors are in the parenthesis. Source: Authors’ calculation.

Table 7 gives a report of the results of the Pedroni panel cointegration test using both of the two models, which include the trend components and the no-trend components. The high values of the Modified Phillips-Perron, Phillips-Perron, and Augmented Dickey-Fuller t-statistics (*p* < 0.05) are evidence in support of the existence of a long-run equilibrium relationship between life expectancy, GDP, health expenditure, carbon emission, urbanization, and trade openness. This implies that there are shifts in these variables that move with time across the BRICS economies. Although economic growth and the investment in healthcare will have long-term positive impacts on life expectancy, the environmental issues and trade-related activities are still going to impact the long-term adjustment dynamics, underscoring how development, health, and environmental pressure have always affected these nations.

**Table 7:** Pedroni cointegration test.

Test		Without trend		With trend	
		Statistics	*p*-value	Statistics	*p-*value
Pedroni	Modified Phillips-Perron t	1.5215	0.0641	1.7098	0.0437
	Phillips-Perron t	−1.9603	0.0250	−2.7546	0.0029
	Augmented Dickey-Fuller t	−1.5588	0.0595	−2.5795	0.0049

Source: Authors’ calculation.

Table 8 indicates the outcome of univariate quantile regression, the impact of the economic, health, environmental, urbanization, and trade openness variables on life expectancy under the conditions of Q0.05–Q0.90. The findings show that the coefficients are very much heterogeneous, meaning that the determinants of longevity are quite different across countries that have low, intermediate, and high levels of life expectancy. This heterogeneity underscores the need to implement policy interventions that are specific to individual countries as opposed to generalizing and assuming that the BRICS nations would have exactly the same effects.

**Table 8:** Quantile regression.

Variable	Q (0.05)	Q (0.25)	Q (0.50)	Q (0.75)	Q (0.90)
LGDP	−0.517506*** (0.0335788)	−0.2813205*** (0.0759933)	−0.2460112*** (0.0643779)	−0.2742573*** (0.0416768)	−0.3810032*** (0.0389415)
LHE	0.1572323*** (0.0105001)	0.0820992*** (0.023763)	0.03662* (0.0201309)	0.007531 (0.0130323)	0.0372243*** (0.012177)
LCO2	0.1869788*** (0.0074646)	0.1126943*** (0.0168934)	0.103132*** (0.0143112)	0.1103592*** (0.0092648)	0.1336499*** (0.0086567)
LUP	0.7267405*** (0.0546902)	0.452916*** (0.1237711)	0.5071051*** (0.104853)	0.6690148*** (0.0678795)	0.8006884*** (0.0634245)
LTOP	−0.0237564** (0.0114881)	−0.1654324*** (0.0259991)	−0.1280037*** (0.0220252)	−0.0751148*** (0.0142586)	−0.0669124*** (0.0133228)
Cons	3.485556*** (0.0881665)	4.118583*** (0.1995325)	3.814623*** (0.1690345)	3.339749*** (0.1094292)	3.361263*** (0.1022471)

*p < 0.1, **p < 0.05, ***p < 0.01. Standard errors are in the parenthesis. Source: Authors’ calculation.

Across the estimated quantiles, GDP (LGDP) shows a statistically significant negative association with life expectancy, with coefficients ranging from −0.517 at Q0.05 to −0.381 at Q0.90. This pattern suggests that higher aggregate output does not uniformly translate into improved longevity outcomes within the BRICS sample, particularly at lower segments of the life expectancy distribution. One possible explanation is that growth episodes accompanied by unequal income distribution, pollution intensive industrialization, and uneven access to healthcare may weaken the expected health gains from rising income. This result contrasts with the conventional positive growth–health relationship widely reported for high income and institutionally advanced economies (30, 83, 84), but is consistent with more recent nonlinear and distribution sensitive evidence showing that the health effects of growth depend on structural and environmental conditions. The finding therefore supports the view that growth quality and inclusiveness matter for health outcomes, and that macroeconomic expansion alone is not sufficient to guarantee longevity improvements.

Health expenditure (LHE) exhibits a positive and statistically significant association with life expectancy across most quantiles, with coefficients declining from 0.157 at Q0.05 to 0.037 at Q0.90. This pattern indicates that increased health spending is generally linked with improved longevity outcomes, particularly at lower levels of the life expectancy distribution. The diminishing marginal effect at higher quantiles suggests that beyond a certain threshold, the efficiency, targeting, and institutional quality of spending become more important than the aggregate volume of expenditure. This distributional behavior is consistent with prior empirical evidence showing that well directed and policy supported health investments contribute to better population health outcomes (37, 39, 85). However, contrasting evidence also exists. Kim and Lane (86) report a negative association across U. S. states, emphasizing that allocation structure and governance quality can outweigh spending levels alone, which aligns with the heterogeneous effects observed here.

Carbon emissions (LCO₂) show a positive association with life expectancy across quantiles, with coefficients ranging from 0.187 to 0.134. This result reflects the dual role of emissions as both an indicator of environmental pressure and a proxy for industrial and economic activity in emerging economies. Similar mixed patterns have been noted in transition stage economies, although many cross country studies find a negative pollution–health relationship overall (87). The finding therefore suggests a development stage effect consistent with Environmental Kuznets type dynamics and highlights the need to coordinate environmental and health policies alongside industrial expansion.

Urbanization (LUP) displays a heterogeneous relationship with life expectancy across quantiles, with a positive coefficient at the lower tail (0.727 at Q0.05) and a negative coefficient at the upper tail (−0.801 at Q0.90). This pattern suggests that urban development may improve longevity where baseline health and service access are relatively low, but its effects can weaken or reverse at higher life expectancy levels when congestion, environmental stress, and infrastructure pressure dominate. The positive lower-quantile effect is consistent with evidence that planned and service-oriented urban expansion enhances access to healthcare, education, and basic infrastructure in developing settings (21, 88). At the same time, the negative upper-quantile association aligns with findings that rapid or poorly managed urbanization can strain health systems and reduce long run health gains (89). These mixed estimates support a conditional interpretation in which urbanization outcomes depend on planning quality and institutional capacity.

Trade openness (LTOP) shows a negative and statistically significant association with life expectancy across all quantiles, with coefficients ranging from −0.024 to −0.167. This indicates that greater external integration may be associated with adverse health outcomes in the BRICS context, possibly through pollution intensive production, lifestyle shifts, and exposure to external shocks. This pattern is broadly consistent with prior evidence highlighting environmental and health tradeoffs linked to liberalized trade in developing economies (23). Related work also reports nonlinear interaction effects in which trade initially amplifies pollution related health risks before adjustment mechanisms emerge (90).

Altogether, the results of the quantile regression emphasize the idea that health outcomes within BRICS countries are the result of a complicated set of economic, social, environmental, and structural influencing factors. Although health spending and urbanization are crucial in improving life expectancy, pressures on the environment caused by out-of-control environmental degradation and unequal distribution of economic benefits and impacts on trade can reduce these advantages. This heterogeneity of the quantiles underscores that policy interventions should be context-specific, which needs to combine sustainable urban planning, investment in specific healthcare, and environmental management.

Table 9 also indicates that the quantile regression analysis with the assistance of other estimators (PCSE, DKSE, and F-GLS) shows a consistent and sufficient pattern throughout the BRICS countries. It seems that GDP negatively influences life expectancy, which may imply that economic growth may not necessarily be accompanied by better health outcomes. Conversely, there is a positive and significant effect of health expenditure, carbon emissions, and urbanization, which explains the significance of investing in healthcare, urban development, and infrastructure for the population’s health. The effect of trade openness is always negative, which indicates the possible health-related dangers of industrialization and globalization. These findings are consistent when compared against various estimation techniques and are indicative of strong validity of the results and the intrinsic multidimensionality of the relationship between economic, environmental, and social variables in determining life expectancy in the BRICS region.

**Table 9:** Robustness check (PCSEs, DKSE, F-GLS).

Variable	PCSEs	DKSE	F-GLS
LGDP	−0.2756096***(0.0388062)	−0.2756096***(0.0626337)	−0.2756096***(0.0445028)
LHE	0.0625705***(0.012557)	0.0625705***(0.0085199)	0.0625705***(0.013916)
LCO2	0.1186184***(0.0082535)	0.1186184***(0.0132928)	0.1186184***(0.009893)
LUP	0.5151382***(0.0552601)	0.5151382***(0.1200055)	0.5151382***(0.0724823)
LTOP	−0.1309885***(0.0180168)	−0.1309885***(0.0170976)	−0.1309885***(0.0152255)
Cons	3.781129***(0.0830425)	3.781129***(0.0945328)	3.781129***(0.1168493)

*p < 0.1, **p < 0.05, ***p < 0.01. Standard errors are in the parenthesis. Source: Authors’ calculation.

Table 10 demonstrates the findings of the Dumitrescu-Hurlin (D-H) causality test and indicates a one-way causality of life expectancy on GDP, health expenditure, and openness to trade. This implies that the better the population health is, the more it is one of the drivers of macroeconomic and structural developments. The only variable with a bidirectional causal relationship with life expectancy is urbanization, which seems to increase with time. The fact that most of the variables are not reversibly causal points to the fact that sustained health gains are critical toward encouraging equitable and sustained economic and social development across the BRICS countries.

**Table 10:** D-H causality test.

Variables relationship	W-stat	Z-bar	*p*-value
LLE → LGDP	11.3463	16.3590	0.0000
LLE → LHE	10.4079	14.8752	0.0000
LLE → LCO2	11.3407	16.3502	0.0000
LLE → LUP	5.4080	6.9697	0.0000
LLE → LTOP	2.2976	2.0516	0.0402
LGDP→ LLE	1.3156	0.4990	0.6178
LHE → LLE	1.1809	0.2861	0.7748
LCO2 → LLE	1.4204	0.6647	0.5062
LUP → LLE	3.5972	4.1065	0.0000
LTOP→ LLE	1.9999	1.5809	0.1139

Source: Authors’ calculation.

## 5 Conclusion and policy recommendations

This study examines the relationships among economic growth, healthcare expenditure, environmental pollution, urbanization, trade openness, and life expectancy in BRICS countries over the period 2000–2024 using a distribution-sensitive panel framework. A set of cross-sectional dependence, slope homogeneity, and unit-root tests confirmed the data’s validity, while panel cointegration supported a stable long-run relationship among the key variables. The quantile regression results show marked heterogeneity across the life-expectancy distribution, suggesting that the effects of the determinants vary across development levels.

Revisiting the initial hypotheses, the findings confirm the positive influence of healthcare spending, partial support for urbanization, mixed results for pollution and growth, and a consistently negative association for trade openness. Several of these relationships differ from conventional theoretical expectations, especially the conditional growth and pollution effects, underscoring the importance of context and distributional differences among the BRICS countries. The results indicate that policy priorities should reflect these differences rather than follow a uniform pattern. Gains in longevity are linked most closely to efficient, well-targeted healthcare expenditure—particularly where life expectancy remains relatively low. Urbanization supports health improvements when accompanied by adequate housing, sanitation, and service infrastructure but can have the opposite effect under overcrowding or environmental stress. Similarly, the interaction between industrial expansion and environmental management appears to depend on each country’s stage of transition, making it essential to coordinate growth strategies with pollution control and clean-technology adoption.

The negative relationship between trade openness and life expectancy points to a more complex dynamic between global integration and population health. Trade liberalization, while expanding economic opportunities, can also increase exposure to environmental degradation, hazardous production processes, and fiscal pressure on health systems if not supported by proper regulation (65, 91). The result does not imply that trade is inherently harmful, but rather that its benefits depend on how integration is managed. Governments should pair trade policies with clear environmental and occupational-health standards, promote cleaner export industries, and protect healthcare budgets from external shocks associated with trade fluctuations. Such measures can help ensure that openness contributes to sustainable, health-secure growth. Overall, the evidence suggests that health and development policies in BRICS countries must be adaptive and context-specific. Strengthening health-system efficiency, investing in pollution mitigation, and embedding social and environmental safeguards in trade and industrial policies would create more balanced and resilient pathways toward longer, healthier lives.

## 6 Limitations and recommendation for future research

First, the research focuses mainly on the amount of healthcare spending rather than the quality, efficiency, and accessibility of healthcare systems, which can differ widely both across and within countries. These unmeasured aspects could affect how health expenditure translates into outcomes. Future research should therefore examine the nature and effectiveness of healthcare systems and delivery models—particularly in low- and middle-income countries—to explore how health spending can be used most efficiently to improve longevity. Second, the analysis is confined to the BRICS countries. While this allows for meaningful comparison among large emerging economies, it limits the generalizability of the findings to other developing or high-income regions that operate under different health systems, economic structures, and institutional settings. Expanding future studies to include a broader group of emerging and developing economies would provide more comprehensive and region-specific insights, helping to assess whether similar patterns hold beyond the BRICS context.

Third, the environmental assessment considers only carbon emissions, whereas other pollutants—such as air, water, and industrial waste—may also influence public health and life expectancy. Including multiple environmental stressors in future analyses would offer a more complete understanding of how ecological factors interact with economic development and health outcomes. Fourth, the study does not account for unexpected global shocks such as pandemics or financial crises, which can have significant short- and long-term impacts on health and life expectancy. Future longitudinal research could evaluate the enduring effects of such shocks, helping policymakers design more resilient health and economic systems. Fourth, as in most macro-panel analyses, reverse causality and other forms of endogeneity cannot be completely ruled out. For instance, while economic growth, healthcare expenditure, and environmental quality may affect life expectancy, improvements in population health can simultaneously promote higher productivity, advances in human capital, and greater fiscal capacity for public spending. Moreover, unobserved variables such as institutional quality or social development may influence both health outcomes and the explanatory variables. Future research could employ instrumental variable techniques, propensity score matching to address these potential endogeneity concerns and better isolate structural effects.

Finally, as with most cross-country panel studies, this analysis may be subject to omitted variable bias resulting from unobserved factors—such as governance quality, social protection measures, or cultural attitudes toward health—that may jointly influence both the explanatory variables and life expectancy. Incorporating additional institutional and social indicators, or employing instrumental variable approaches, would help address potential biases and strengthen the validity of the findings. Overall, future research could extend this line of inquiry by comparing healthcare performance across different regions, integrating broader environmental and institutional variables, and analyzing the long-term and shock-responsive effects of health and economic policies. Such approaches would provide stronger evidence on how economic, social, and environmental conditions jointly shape population wellbeing over time.

## Data Availability

The original contributions presented in the study are included in the article/supplementary material, further inquiries can be directed to the corresponding author.
